# Assessment and Treatment of Pain during Treatment of Buruli Ulcer

**DOI:** 10.1371/journal.pntd.0004076

**Published:** 2015-09-24

**Authors:** Janine de Zeeuw, Marike Alferink, Yves T. Barogui, Ghislain Sopoh, Richard O. Phillips, Tjip S. van der Werf, Susanne Loth, Bouwe Molenbuur, Mirjam Plantinga, Adelita V. Ranchor, Ymkje Stienstra

**Affiliations:** 1 University of Groningen, University Medical Center Groningen, Department of Internal Medicine/Infectious Diseases, Groningen, The Netherlands; 2 University of Groningen, University Medical Center Groningen, Department of Health Psychology, Groningen, The Netherlands; 3 Programme National de Lutte contre la Lèpre et l´Ulcère de Buruli, Ministère de la Santé, Cotonou, République du Bénin; 4 Kwame Nkrumah University of Science and Technology, School of Medical Sciences, Department of Medicine, Kumasi, Ghana; 5 University of Groningen, University Medical Center Groningen, Department of Anesthesiology, Groningen, The Netherlands; 6 University of Groningen, University Medical Center Groningen, Department of Genetics, Groningen, The Netherlands; Fondation Raoul Follereau, FRANCE

## Abstract

**Background:**

Buruli ulcer (BU) is described as a relatively painless condition; however clinical observations reveal that patients do experience pain during their treatment. Knowledge on current pain assessment and treatment in BU is necessary to develop and implement a future guideline on pain management in BU.

**Methodology:**

A mixed methods approach was used, consisting of information retrieved from medical records on prescribed pain medication from Ghana and Benin, and semi-structured interviews with health care personnel (HCP) from Ghana on pain perceptions, assessment and treatment. Medical records (n = 149) of patients treated between 2008 and 2012 were collected between November 2012 and August 2013. Interviews (n = 11) were audio-taped, transcribed verbatim and qualitatively analyzed.

**Principal Findings:**

In 113 (84%) of the 135 included records, pain medication, mostly simple analgesics, was prescribed. In 48% of the prescriptions, an indication was not documented. HCP reported that advanced BU could be painful, especially after wound care and after a skin graft. They reported not be trained in the assessment of mild pain. Pain recognition was perceived as difficult, as patients were said to suppress or to exaggerate pain, and to have different expectations regarding acceptable pain levels. HCP reported a fear of side effects of pain medication, shortage and irregularities in the supply of pain medication, and time constraints among medical doctors for pain management.

**Conclusions:**

Professionals perceived BU disease as potentially painful, and predominantly focused on severe pain. Our study suggests that pain in BU deserves attention and should be integrated in current treatment.

## Author Summary

Buruli ulcer (BU) is considered relatively painless. Nevertheless, observations suggested that patients experience pain during wound care dressings. This study explored views on pain, along with pain assessment and treatment practices. Medical records were reviewed on prescribed pain medication and health care professionals involved in BU treatment were invited for an interview to elicit their views on pain including current pain practices. Interviews were held in private locations, audio-taped, and analyzed qualitatively. In the majority of medical records, pain medication was prescribed. Mostly simple analgesics were prescribed, while health care professionals reported not being trained in the assessment of mild pain, and indications were often missing. Health care professionals indicated advanced BU might be painful, and that pain can increase after wound treatment, and after a skin graft at the donor site. They perceive the recognition of pain as difficult as patients suppress or exaggerate pain, and have different expectations regarding acceptable pain levels. Finally, they indicated a fear of side effects of pain medication, a shortage of, and irregularities in supply of pain medication, and limited time among medical doctors for pain management. These findings indicate pain during BU disease deserves attention and pain practices should be integrated in standard treatment.

## Introduction

Buruli ulcer (BU) is a Neglected Tropical Disease for which the World Health Organization (WHO) stressed the need to improve treatment [1]. BU remains endemic in areas in West Africa, especially in Benin and Ghana [2,3]. In Benin, prevalence rates between 5.4 and 60.7 per 10.000 inhabitants have been reported each year [4] while in Ghana, prevalence rates have fluctuated between 2.0 and 15.0 per 10.000 inhabitants [5]. BU destroys skin, subcutaneous fat, and sometimes bone [6]. Patients typically present with non-ulcerated lesions; papules, nodules, plaques or edema, or undermined ulcers [6,7]. Treatment entails antibiotics complemented with surgery if needed, together with dressing changes of which the frequency depends on the wound, and physiotherapy [8]. Specialized treatment centers deliver the necessary care while antimicrobial treatment and dressings may also be delegated to local health centers [9]. Irrespective of therapy (antibiotics or surgery), 47% of patients are left with functional limitations after healing [10].

Although BU is described as relatively painless [11–13] clinical observations reveal that at least some patients experience considerable pain during their treatment—especially during wound care and physiotherapy. This is supported by two studies, i.e., one in Japanese patients [14], and one in Ghanaian patients [15]. Ghanaian BU patients sometimes complained of pain at the lesion site just before and during ulceration. This suggests a recovered sensation—perhaps due to a decrease in mycolactone concentrations [16] in tissues at advanced disease stages, however this suggestion warrants verification by clinical studies. Clinical studies on pain and pain treatment among BU patients are lacking. The recognition, management and treatment of pain is a challenge worldwide, but limited resources in low- and middle-income countries further increase the risk of under treatment of pain [17,18]

Health care personnel (HCP) treating BU in Ghana and Benin stressed the need to include pain management [19]. The WHO recommends integrating pain treatment as part of general treatment of patients globally [18]. Pain management should follow a practice guideline, which is currently not available for BU treatment, including at least an analgesic ladder, i.e. the ‘pain ladder’, initially developed by the WHO originally for cancer pain control [20,21], but now used for pain relief among patients with wound pain [22]. The ladder consists of three steps of drugs with increasing analgesic effects.

Previous studies showed that perceptions and beliefs of health professionals are important in pain treatment [23]. In addition, barriers related to the availability of- and access to analgesics, a lack of education and information among HCP, and legal barriers such as regulatory restrictions on opioids exists, preventing effective pediatric pain treatment in sub-Saharan Africa [24]. Furthermore, language barriers, fear of addiction to opioids, and cultural differences between patients and health professionals, influenced pain management in Central Africa [25].

Firstly, this study investigates current pain practices in BU in Ghana and Benin, including the prescription of pain medication, and the use of the WHO pain ladder by the HCP. Secondly, this study aims to explore factors that might influence the success of a possible future guideline implementation.

## Methods

### Design and participants

A mixed methods approach was used, consisting of information on prescribed pain medication, retrieved from medical records from Ghana and Benin, and semi-structured interviews. Data collection was performed in the same period in both countries. Two out of the four BU treatment centers in Benin, and two out of the four BU treatment centers in Ghana were selected for the study. Information from the medical records of patients treated between 2008 and 2012 was collected between November 2012 and August 2013. In the two treatment centers in Ghana, medical records of all PCR confirmed BU patients admitted and treated in the selected time period, were included (n = 69). In the two treatment centers in Benin, a larger number of records of PCR confirmed BU cases was found in the selected time period, thus a systematic selection (every *8* ^th^ medical record) was performed in both centers to arrive at 40 records per center. Thus, in Ghana 69 medical records of patients were included, and in Benin 80 medical records of patients leading to a total of 149 patients. Eligible HCP were selected by purposive sampling based on their involvement in BU wound care, their profession—we selected medical doctors, nurses, physiotherapists and local health workers, to ensure heterogeneity of the interviewees-, and the ability to speak English. In total 13 HCP were eligible and approached. Interviews were conducted between August 2012 and May 2013.

### Data collection

The interviews were held in private settings in the hospital or health center in Ghana, and were conducted by one of the authors (MA, JDZ or SL). Interviews were tape recorded and lasted between 60 and 90 min.

### Materials

Data on general characteristics and diagnostic tests were collected from the records. The type of pain medication and its indication were retrieved as well. An interview topic guide was developed, covering HCPs perceptions on current pain assessment and treatment. Specific topics included were; current practice, current prescription of pain medication, HCPs preferences for prescribing pain medication, satisfaction with current practice, wishes for improvements, perceptions on pain, pain assessment, and the acceptability of showing pain. Questions were derived from previously published work [24]. Probes were used where necessary. Face and content validity of the interview guide were assessed by the study team, ensuring that questions adequately covered the objectives and were relevant.

### Data analysis

Data on prescribing behavior and patient characteristics from the records were analyzed using Statistical Package for Social Science version 20. Descriptive analysis was performed on age and sex of patients whose records were included, including the type of lesion, indication for pain medication, types of pain medication prescribed during hospitalization and the types of pain medication prescribed in line with the WHO pain ladder. The total number of times that pain medication was prescribed for each step of the WHO pain ladder was counted. Interviews were transcribed verbatim by three different researchers (MA, JDZ and SL). A qualitative content analysis was performed using open coding and axial coding. To ensure reliability, interviews were analyzed consecutively by two researchers (MA and JDZ). These two researchers developed an initial codebook independently using one interview, and taking into account the interview questions. Both researchers participated in weekly meetings to extensively discuss the open coding analysis until consensus was reached, after which codes were merged into one codebook. The codebook was adapted throughout the analysis, based on new codes emerging from the data. A third and fourth researcher (AVR, YS) were consulted in case of disagreement, and ensured an acceptable coding of the data, ordering of the codes, and selection of themes. Qualitative and quantitative data were combined according to parallel data analysis [26].

### Ethics

Ethical clearance was obtained from the Medical Ethical Review Committee of the Kwame Nkrumah University of Science and Technology; School of Medical sciences, Komfo Anokye teaching hospital in Ghana (ref: CHRPE/AP/230/12). Data of medical records was handled in line with Good Clinical Practice. Written informed consent was obtained from all HCP. Before the interview started, voluntary participation, confidentiality, the aim, the topic and type of questions, the rights to withhold certain information, and to withdraw during the interview, were explained. Written informed consent was obtained from all HCP. In order to ensure anonymity and confidentiality, a number was assigned to each interview.

## Results

### Prescribed pain medication

#### Patient characteristics from medical records

In total, 149 medical records were reviewed (69 from Ghana, 80 from Benin), of which 14 were incomplete, resulting in 135 records included in the analysis. In 113 (84%) of the 135 included records, pain medication was prescribed at least once during hospitalization. The median weeks of hospitalization was 17 (IQR: 10–23). The median age of the patients receiving pain medication was 14 years (IQR: 8–28), and 65 (58%) was male (Table 1). In 64% of the medical records, ulcers were reported, 15% plaque, 10% edema and 3% nodules (8% missing).

**Table 1:** Patient characteristics (n = 113).

		Total
Sex (male)		65 (58%)
Age (Median, IQR)		14 (8–28)
Type of ulcer	Ulcer	64%
	Plaque	15%
	Edema	10%
	Nodule	3%
	Not reported	8%
Duration of hospitalization in weeks (Median, IQR)		17 (10–23)

#### Type of pain medication (different generics) prescribed during hospitalization

In most records (43.4%; n = 49), two types of pain medication were prescribed, while in 35.4% of records (n = 40), one type of pain medication was prescribed. In 13.3% of the records (n = 15), three types were prescribed and in 7.9% of the records (n = 9), four or five types of medication were prescribed.

#### Pain medication according to WHO ladder

The total number of times pain medication was prescribed was 482. Step 1 medication (i.e. simple analgesics) was mostly prescribed (n = 430), for several indications. Pain medication is mostly prescribed after a surgical intervention (n = 165; 34%) (Table 2), however, often, no indication was noted in the records (n = 233; 48%). Step 2 and 3 medications (weak and strong opioids, respectively) were rarely prescribed (n = 52) (Table 2). Predominantly, pain medication is prescribed after an intervention, and less often for background pain.

**Table 2:** Pain medication prescribed along different indications according to WHO ladder (n = 482).

Indication	Step 1*	Step 2**	Step 3***
Post-surgical pain	151	12	2
(before) wound dressing	5	5	0
Other interventions (e.g. removal of necrotic tissue)	14	3	0
Infected wound/wound related pain	12	2	0
Generalized body pain	18	1	0
Not related to pain (e.g. fever, diarrhea)	22	2	0
Missing indication	208‡	24	1
**Total**	**430**	**49**	**3**

*Paracetamol/acetaminophen, ibuprofen, acetylsalicylic, diclofenac and diclofenac sodium.

** paracetamol/codeine, tramadol and dihydrocodeine.

*** pethidin ‡ of the missing indications 136 times paracetamol/acetaminophen was prescribed

### Current pain assessment and treatment-results from the semi-structured interviews-

11/13 eligible HCP agreed to participate in the semi structured interview; nurses (4), medical doctors (2), BU coordinators (2), physiotherapist (1), health worker (1) and pharmacy technologist (1). Two participants refused because of a lack of time. Three factors in current pain assessment and treatment were important, i.e., perceptions on pain, pain assessment and pain treatment, including the use of the WHO pain ladder.

### Perceptions on pain in BU

Pain is described as ‘*an uncomfortable feeling*’ or ‘*an alarm*’. In early stages of the disease, BU is painless, however, in later stages patients start to feel pain: ‘*So when they start to heal*, *they start to feel pains’*.

Perceived causes of pain were; wound pus, ulceration of the lesion, inflammation, nerve exposure, secondary infection, a graft at the donor site, proximity to bone or joint. An increase in pain is mentioned after wound treatment.

*‘Yes*, *normally*, *patients with BU*, *actually don’t have pain*. *Unless there is a secondary bacterial infection*. *There is involvement of the nerves and involvement of the bone tissue’*.

Pain threshold is described by the HCP as ‘*The extent to which a person can handle pain*’, or as ‘*How patients react to pain’*. According to the participating HCP, patients differ in their pain threshold and pain tolerance. Factors related to differences in pain tolerance are; previous experiences, gender differences, age of the patient, and size of the ulcer.

### Expression of pain and its assessment

Nurses and a physiotherapist reported not to be trained in assessing mild pain, neither do they ask patients about mild pain. They attributed this to their culture; patients are able to handle mild pain.

‘*We [HCP] are not trained to recognize the mild ones quickly*, *it is a cultural problem*, *we know people take the mild ones*, *so I don’t ask*, *suggest pain to you [patient]*.*’*

Instead of focusing on whether patients report pain, professionals pay attention to facial expression, body language, wound characteristics, individual characteristics, medical background, and patients’ behavior.

*‘But you can see from the face whether it is painful or not*. *So we look for signs*, *because otherwise*, *we are going to get wrong feedback*.’

*‘Assuming you have a lesion*, *and I touch it*, *and you don’t complain*, *that means you are ok*, *there is no pain*. *But if I touch it [the lesion] and you pull your hand*, *I mean the little thing I do*, *hurts to your hand*, *and you are preventing me*. *The moment you start and the patient is starting crying and screaming and all these things*, *you can easily know that now*, *the patient has a severe pain*.*’*

Suppressing pain expression is ascribed to cultural factors; which is especially common among adult males, patients from the northern part of the country, patients from rural areas, and patients with a lower educational level. This cultural tendency hampers a proper pain assessment.

*‘Location wise*, *you see people from the northern part of the country*, *they are very good adapted at taking pain*. *I would rather classify them as the affluent compared to the less affluent or the region poor or urban and the village*. *If it is in the city*, *well-educated parents know that they shouldn’t be treated this way*, *so they get better treatment when you have more enlightened parents*. *People try to contain pain*. *It is a country wide a cultural thing*, *we are brought up to be able to contain an amount of pain*, *so you are a cry baby if you express too much pain*.*’*

On the other hand, the majority of the HCP mentioned that patients exaggerate pain. This paradox could imply that—since HCP are part of the Ghanaian culture in which suppression of pain expression is commonly seen—they believe that patients who express pain overtly, are exaggerating.

*If it is true pain*, *or fake pain*, *we don’t know*, *you measure*, *you look at the patient*.*’*

*‘You rate it for the patient*, *but not what the patient tells you*. *Because psychologically*, *the patient would tell you ‘oh*, *it is very painful’*. *Meanwhile*, *it is not like that*.*’*

### Pain treatment

Important factors in current pain treatment include the financial constrains that patients often have to pay for pain medication. Moreover, HCP report a fear of side effects of pain medication, a fluctuation in availability of pain medication, and a shortage of time among medical doctors.

*‘We shouldn’t over rush in giving pain killers*, *because there are side-effects attached to the drugs’*.

Furthermore, HCP report a discrepancy between their own as compared to their patients’ expectations regarding pain relief. While professionals expect patients to endure pain to some extent, patients expect to be relieved from pain during hospital admission, according to the HCP.

*‘They [patients] believe as soon as they’re in the hospital*, *everything must be stopped*. *As soon as they’re operated they should have no pain at all*, *even not the wound pain*. *I always tell them that operation does not mean that everything is healed*, *that pain stopped*, *the wound is there*, *so it pains’*.

Besides, non-pharmacological factors in the current pain treatment include the different coping strategies used by the HCP to help the patient to handle pain. Examples are: counseling (providing information, reassurance, showing empathy) and giving advice.

*‘Explaining to patient*, *oh no*, *this is just a little thing that I would just only have to give you*, *but it will minimize the pain that you are going through*, *because you fell from the bike*. *So your pain will minimize*.*’*

Furthermore, while the HCP were not explicitly familiar with the WHO ladder, they reported to use the basic principles of the tool.

‘*It is about*, *if someone is in pain then you start with paracetamol and then if someone has more severe pain you can give paracetamol and another pain killer which is more effective and then in the end you can give something like pethidin*.*‘*

## Discussion

This study aimed to explore current pain practice in BU in Ghana and Benin. For most BU patients, pain medication was prescribed, and pain management mainly focused on severe pain. Professionals perceived later stages of BU as painful, and reported an increase in pain after wound treatment, and after a skin graft at the donor site. HCP reported a suppressed pain expression as well as exaggeration in patients, and differences in expectations between professionals and patients on what is an acceptable pain level without medication.

Mainly WHO step 1 pain ladder medication was prescribed, while strong opioids were hardly prescribed. Explanations provided by the HCP on the mild prescriptions were; fear of side effects of strong opioids, fluctuation of availability of pain medication, and the shortage of time among medical doctors. These findings are in line with literature mentioning resistance among HCP to use morphine [27]. Alternative explanations are that HCP did not ask whether patients experienced pain, or a lack of attention for mild pain. At the same time, the prescribed pain medication is typically for mild pain, according to the WHO pain ladder indicating that severe pain is treated with mild pain medication.

It appeared that the indication for pain medication was often not documented in the medical records. Effective pain relief ultimately depends on accurate pain assessment, and the nature, severity, location and duration of pain should be assessed and documented to understand the possible etiology and to effectively treat pain [28].

Both the finding that HCP believed that patients exaggerated their pain, and the expectation that patients should endure pain, might be influenced by sociocultural factors, since pain expression varies across cultures worldwide [28]. In different African ethnic groups, stoicism is a valued response to pain [29,29,30]. Despite the cultural factors, differences in pain expression can be due to individual differences [31]. An important implication of our finding is that it complicates pain assessment for professionals.

Patients and professionals differed in their expectation on acceptable pain levels without medication. HCP noticed that patients expected to be pain free during hospitalization, a statement that should be confirmed in studies using a patients’ perspective. If these results can be confirmed, patients’ expectations could be addressed during the preoperative pain assessment by collaboratively setting goals for pain control [32], or during the intake for hospitalization. Furthermore, if documentation of pain will be integrated in daily practice, pain can be monitored, which is essential for adequate treatment.

This study had several limitations. For the part on the medical records, an information bias might have occurred due to the retrospective design of the study. In a small proportion of medical records, no information on pain medication was found, and in case pain medication was prescribed, the indication was often missing. In addition, by using medical records, only the prescribing behavior of HCP was studied, while the actual intake remained unknown. A limitation of the interviews was the possible bias introduced by the role of non-native interviewers. Although the interviewers spent time in the different BU settings, cultural differences remained. This might have influenced the interviews.

To conclude, these findings, together with a study on wound care-related pain in BU [15], suggest that there is room for improvement to arrive at adequate pain treatment. Several factors could be taken into account when developing a pain guideline, such as the current practice on prescribing pain medication, the discrepancy between HCP and patients about pain relief, the views on pain expression and suppression, recognition and treatment of mild pain, and the lack of recorded indications. Furthermore, the findings that HCP tried to help the patient to cope with pain by providing information, reassurance, showing empathy, giving advice, as well as the finding that HCP were aware of the basic principles of the WHO ladder are useful in the development phase of such a protocol.
